# Lymphatic vessels help mend broken hearts

**DOI:** 10.7554/eLife.52200

**Published:** 2019-11-11

**Authors:** Catherine Pfefferli, Anna Jaźwińska

**Affiliations:** Department of BiologyUniversity of FribourgFribourgSwitzerland

**Keywords:** cardiac lymphatics, regeneration, zebrafish, revascularization, cryoinjury, Zebrafish

## Abstract

Experiments on zebrafish show that the regeneration of the heart after an injury is supported by lymphatic vessels.

**Related research article** Harrison MRM, Feng X, Mo G, Aguayo A, Villafuerte J, Yoshida T, Pearson CA, Schulte-Merker S, Lien CL . 2019. Late developing cardiac lymphatic vasculature supports adult zebrafish heart function and regeneration. *eLife* **8**:e42762. doi: 10.7554/eLife.42762

**Related research article** Gancz D, Raftrey BC, Perlmoter G, Marín-Juez R, Semo J, Matsuoka RL, Karra R, Rviv H, Moshe N, Addadi Y, Golani O, Poss KD, Red-Horse K, Stainier DYR, Yaniv K . 2019. Distinct origins and molecular mechanisms contribute to lymphatic formation during cardiac growth and regeneration. *eLife* **8**:e44153. doi: 10.7554/eLife.44153

The heart, like any other organ, is spanned by a network of blood and lymphatic vessels (Petrova and Koh, 2018). The importance of blood vessels to the heart is obvious, but what about the lymphatic conduits, which are best known for carrying immune cells from tissues to the lymph nodes, where immune responses can be triggered? As it happens, lymphatic vessels have an important role in helping the heart to recover from injury. When the heart of a mammal is injured, it cannot regenerate, and it scars instead. However, lymphatic vessels in the heart help the healing process by removing inflammatory fluid from the wounded tissue (Norman and Riley, 2016). Thus, the lymphatic system limits swelling and fibrosis (scarring), both of which can interfere with heart function. Until recently it had been difficult to image the lymphatic capillaries within heart tissue, but the discovery of genes that are specifically expressed in them has helped researchers to overcome this problem (Semo et al., 2016).

The zebrafish is a vertebrate with an extraordinary capacity to regenerate its organs, including the heart. However, relatively little is known about the role of lymphatic vessels in the regeneration of the zebrafish heart. Now, in eLife, Michael Harrison (Saban Research Institute of Children’s Hospital Los Angeles), Ching-Ling Lien (Saban and University of Southern California), and co-workers in the United States and Germany report the results of experiments that explore what role, if any, lymphatic conduits have in the regeneration of the zebrafish heart (Harrison et al., 2019). To do this the researchers genetically modified zebrafish so that their blood and lymphatic vessels were fluorescent, thus allowing both sets of vessels to be tracked over time (Semo et al., 2016; Hogan and Schulte-Merker, 2017).

In the zebrafish heart, under normal conditions, lymphatic growth depends on coronary vessels. The coronary arteries develop first, and then provide guiding cues for the lymphatic vessels, which migrate from the outflow chamber of the heart towards the apex of the ventricle (Figure 1A). Indeed, fish that lack coronary arteries cannot form cardiac lymphatic conduits: in these fish, lymphatic vessels are present at the outflow chamber but they cannot spread down over the ventricle. Thus, the formation of the lymphatic vasculature on the heart surface requires coronary arteries.

**Figure 1.**
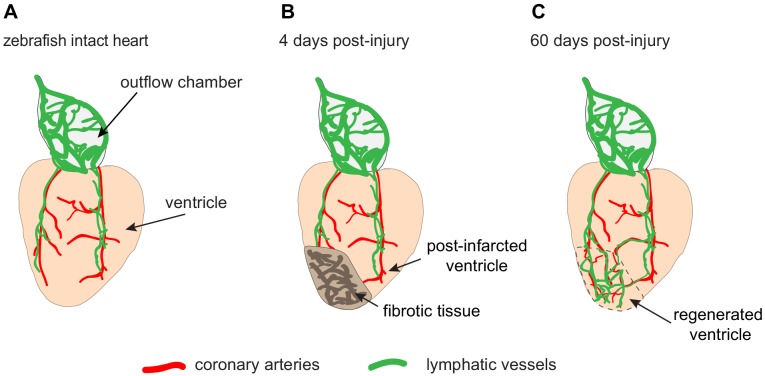
The lymphatic vessels of the zebrafish heart facilitate regeneration after injury. (**A**) Schematic drawing showing the ventricle (pale orange) and outflow chamber (pale gray) of an adult zebrafish heart. The outer surface of the outflow chamber contains many large lymphatic vessels (green), and the ventricle is spanned by a few coronary arteries (red) that guide lymphatic vessels during growth. (**B**) Four days after traumatic cryoinjury, fibrotic tissue has accumulated in the wounded tissue, and this area becomes densely vascularized during the healing process. (**C**) At 60 days post-injury, fibrotic tissue has been replaced with a regenerated cardiac muscle (enclosed within the dashed line). Newly formed lymphatic vessels facilitate this regeneration by removing excessive fluid and inflammatory cells from the damaged tissue.

Harrison et al. asked how lymphatic vessels react to heart damage in zebrafish. They applied two methods of traumatic injury commonly used in regeneration studies: resection and cryoinjury (reviewed in Jaźwińska and Sallin, 2016 and González-Rosa et al., 2017). Resection involves the complete removal of tissue from the heart. In cryoinjury, the myocardial wall is destroyed by freezing it, but the frozen tissue remains attached to the heart: this means that healing involves a considerable amount of inflammation to clear away cellular debris. Nevertheless, after both types of injury, the zebrafish heart can completely regenerate within one to two months.

Harrison et al. analyzed whether heart regeneration is accompanied by the regrowth of lymphatic vessels. They observed that more lymphatic vessels formed after cryoinjury than after partial resection (Figure 1B and C). This could be explained by the role of lymphatic vessels in removing fluid and inflammatory cells from the damaged tissue. Harrison et al. showed the importance of these vessels by blocking a lymphatic growth pathway, which resulted in reduced heart regeneration after cryoinjury.

In similar but independent work, Karina Yaniv (Weizmann Institute) and co-workers in Israel, the United States and Germany – including Dana Gancz as first author – report that the lymphatic vessels of the ventricle derive from diverse tissues (Gancz et al., 2019). Most come from the pre-existing lymphatic branches (which expand through sprouting). However, new lymphatic structures can also be formed de novo through the differentiation of progenitor cells resident in the ventricular wall. The formation of the new lymphatic vessels and structures seems to follow the demand for fluid drainage from the ventricular tissue, which increases following myocardial damage. Thus, lymphatic vessels in the heart create an environment favorable to scar resolution and regeneration (which is consistent with a recent study by Vivien et al., 2019).

In zebrafish, the mechanisms of fluid drainage through the heart are still poorly understood. For example, we still do not know how the optimal density of lymphatic vessels is regulated to meet physiological needs, or the cellular origin of newly formed lymphatic clusters. Furthermore, it will be important to test if increasing the rate at which lymphatic vessels are formed will accelerate regeneration. The findings of Harrison et al. and Gancz et al. in the zebrafish may provide clues as to how to manipulate lymphatic vessels to help repair damaged hearts in organisms that do not regenerate. Indeed, even in mammals with no regenerative ability these vessels have been shown to improve tissue repair in pathological conditions such as myocardial infarction (Klotz et al., 2015; Brakenhielm and Alitalo, 2019). Further research into the roles of lymphatic system may lead to important advances in therapies to heal damaged tissues.
